# A frailty index to predict the mortality risk in a population of senior mexican adults

**DOI:** 10.1186/1471-2318-9-47

**Published:** 2009-11-03

**Authors:** José Juan García-González, Carmen García-Peña, Francisco Franco-Marina, Luis Miguel Gutiérrez-Robledo

**Affiliations:** 1 Unidad de Investigación Epidemiológica y en Servicios de Salud, Área Envejecimiento, Centro Médico Nacional Siglo XXI, Instituto Mexicano del Seguro Social. México City. Mexico; 2 Instituto Nacional de Enfermedades Respiratorias. Institutos Nacionales de Salud. México City, México; 3 Instituto de Geriatría. Institutos Nacionales de Salud. México City, México

## Abstract

**Background:**

Frailty in the elderly can be regarded as nonspecific vulnerability to adverse health outcomes, caused by multiple factors. The aim was to analyze the relationships between the frailty index, age and mortality in a two year follow up study of Mexican elderly.

**Methods:**

A frailty index was developed using 34 variables. To obtain the index, the mean of the total score for each individual was obtained. Survival analyses techniques were used to examine the risk ratios for the different levels of the frailty index. Kaplan-Meier estimates were obtained, adjusted for age and gender. Cox proportional hazards models were also built to obtain hazard ratio estimates.

**Results:**

A total of 4082 participants was analyzed. Participants had an average age of 73 years and 52.5% were women. On average, participants were followed-up for 710 days (standard deviation = 111 days) and 279 of them died. Mortality increased with the frailty index level, especially in those with levels between .21 to .65, reaching approximately 17% and 21%, respectively. Cox proportional hazards models showed that participants with frailty index levels associated to increased mortality (.21 and higher) represent 24.0% of those aged 65-69 years and 47.6% of those 85 and older.

**Conclusion:**

The frailty index shows the properties found in the other studies, it allows stratifying older Mexican into several groups different by the degree of the risk of mortality, and therefore the frailty index can be used in assessing health of elderly.

## Background

Frailty in the elderly can be regarded as nonspecific vulnerability to adverse health outcomes, caused by multiple factors [1]. Although in the literature there are many other operationalizations of a frailty definition [2-7], two of them have been widely used. One approach, proposed by Fried et al, considers frailty a distinct clinical syndrome not synonymous with either co morbidity or disability [8]. Under this perspective, frailty is present when an individual has three of the following criteria: unintentional weight loss (10 lbs in past year), self-reported exhaustion, weakness (grip strength), slow walking speed, and low physical activity. Additionally, an intermediate status (prefrailty) is also identified when the individual presents one or two of the above criteria. The other approach, proposed by Mitnitski and Rockwood [9], considers frailty as a continuum of accumulation of deficits (symptoms, signs, diseases, and disabilities) that are combined in a frailty index score, reflecting the proportion of potential deficits present in a person [10].

Apart from the conceptual differences, the two approaches differ in terms of measurement conditions. Fried's approach requires direct measurement of the proposed criteria by health personnel, whereas Mitnitski and Rockwood's approach relies on self report of the presence of deficits. Yet, both approaches have been successfully used in identifying elderly vulnerable to a range of adverse health, outcomes including mortality [1,8].

Three studies have compared both approaches in the same study sample in identifying elderly at high risk of death [11-13] and have found correlation between them, with the deficit accumulation approach predicting mortality better [11,12].

In this paper, we present an analysis of a two year follow up study of Mexican elderly using the deficit accumulation approach to predict mortality.

## Methods

### Study sample

Data analyzed here come from The Mexican Health and Aging Study (MHAS), a prospective panel survey conducted by researchers from the Universities of Pennsylvania, Maryland, and Wisconsin in the U.S., and the Instituto Nacional de Estadística, Geografia e Informática (INEGI) in Mexico [14]. Participants and their spouse/partners were selected from a nationally representative sample of non-institutionalized Mexicans aged 50 and older who had previously participated in the fourth quarter of 2000 in an employment survey (Encuesta Nacional de Empleo, ENE), totaling 15,186 persons. The baseline survey was conducted in the second quarter of 2001 with a second wave conducted approximately two years later.

Individuals were directly interviewed unless they were in poor health or temporarily absent in which case a shorter questionnaire was administered to a proxy. Here we analyze only directly interviewed participants, aged 65 and older.

### Frailty index construction

MHAS questions providing information on the presence of deficits associated to frailty and similar to those used in other studies [9,15-17] were initially identified. Deficits included fell into the following categories: Self-rated health status, medically diagnosed conditions, depression, falls and fractures in the past 2 years, hearing and vision impairments, medical symptoms during the past 2 years, and limitations in activities of daily living and in instrumental activities of daily living. We also included several items available in the MHAS questionnaire pertaining health problems before age 10 which have not been included in the frailty index in previous studies. On the other hand, no questions were included in the MHAS questionnaire on the following items that have been included in the frailty index in previous studies: report of rest tremor, sleep, walking and vibration perception disorders [9], presence of delirium, nutritional [16], dental and skin problems [15].

We used 34 variables to construct the frailty index, following in each of them the scoring suggested by Rockwood et. al. [18]. Most of these variables indicated the presence/absence of a particular deficit, and a score of 1 was given for the presence and a score of 0 otherwise. Hearing and vision problems and body pain were queried through Likert-type scales, with integer values from better to worse starting in 0, and were scored by dividing the participants value by the maximum value in the scale. So, for instance, self-rated health status was scored in the following way: excellent = 0, very good = 0.25, good = 0.50, fair = 0.75 and poor = 1. The depression variable was constructed in a similar fashion after counting the number of depressive symptoms present in the individual (9 questions). Body pain and difficulty with walking were constructed as the depression variable using several MHAS questions. Frailty index was therefore defined as a proportion of the total number of deficits an individual has with respect to the 34 deficits included.

### Statistical analysis

The analyzed MHAS database contained sample weights which were incorporated into statistical procedures using Stata, version 9 [19], when estimates for the Mexican older adult population are presented.

Survival techniques were used to analyze the influence of the different frailty strata on survival. Follow-up time was measured in days. Cumulative mortality Kaplan-Meier estimates were obtained for an average analyzed participant with respect to age and gender. To obtain frailty index strata with minimum variance within observations in a particular stratum and maximum variance between strata, five frailty index levels were determined by a minimum variance method proposed by Dalenius [20]. Cox proportional hazards models were also fitted to analyze the simultaneous influence on survival of frailty index level, age measured in years and gender.

## Results

**Figure 1:**
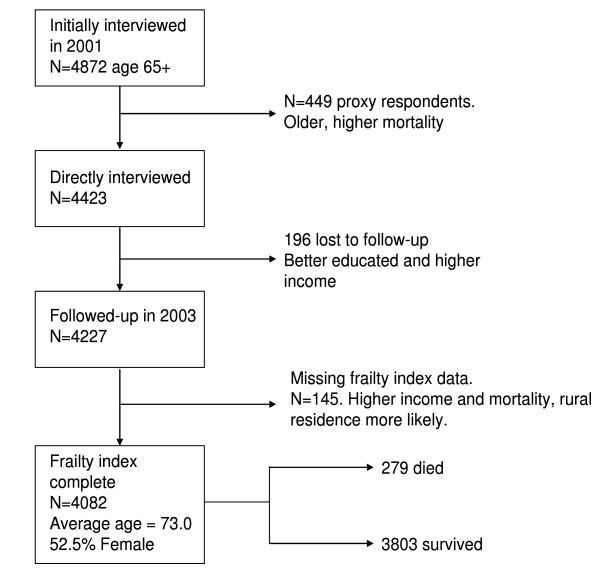
**Mexican Health and Ageing Study (2001) analyzed sample**.

Figure 1 summarizes the characteristics of the analyzed sample. A total of 4872 persons aged 65 and older were interviewed in the MHAS 2001 wave. Of them, we excluded 449 persons (9.2%) because they were not directly interviewed. On average they were 2.6 years older and had a three-fold higher mortality compared to those directly interviewed. In addition, 196 (4.4%) of the 4423 interviewed participants were lost to follow-up. Compared to those not lost to follow-up, not followed-up participants were slightly more educated (1 school year more on average), and had a higher income (2.3 times on average). Finally, after excluding 145 of those followed-up (3.4%) because of missing frailty index data, we analyzed information on 4082 participants (92.2% of those directly interviewed). Analyzed participants had an average age of 73 years (range 65 to 105), with 52.5% being women, both of these figures being very close to the Mexican population aged 65 years and older. On average, analyzed participants were followed-up for 710 days (standard deviation = 111 days) and 279 of them died during the follow-up.

Coding of the health deficits composing the frailty index are shown on table 1, along with their sample weighted mean value, which can also be interpreted as the prevalence of a particular deficit. The most prevalent were vision and hearing problems, leg pain on walking, high blood pressure, falls in the past two years and depressive symptoms with average values between .4 and .5. On the other hand, Cancer and most health problems before age 10 showed the lowest prevalence in the elderly Mexican population with average values below .02.

**Table 1:** Health deficits included in the frailty index and their coding.

**Variable**	**Values**	**Mean†**
**Health problems before age 10**		
1. Tuberculosis	Yes = 1, No = 0	0.009
2. Rheumatic fever	Yes = 1, No = 0	0.015
3. Poliomyelitis	Yes = 1, No = 0	0.007
4. Typhoid fever	Yes = 1, No = 0	0.007
5. Serious head injury	Yes = 1, No = 0	0.049
6. Serious heath problem lasting at least one month	Yes = 1, No = 0	0.125
**General Health**		
7. Poor self-assessed health	Poor = 1, Fair = .75, Good = .5, Very Good = .25, Excellent = 0	0.049
**Medically diagnosed conditions**		
8. High blood pressure	Yes = 1, No = 0	0.438
9. Diabetes mellitus	Yes = 1, No = 0	0.179
10. Cancer	Yes = 1, No = 0	0.014
11. Chronic obstructive pulmonary disease	Yes = 1, No = 0	0.087
12. Heart attack	Yes = 1, No = 0	0.052
13. Stroke	Yes = 1, No = 0	0.039
14. Arthritis/Rheumatism	Yes = 1, No = 0	0.274
Other health problems		
15. Falls in the past two years	Yes = 1, No = 0	0.432
16. Fractures after age 50	Yes = 1, No = 0	0.178
17. Vision problems	Legally blind = 1, Poor = .8, Fair = .6, Good = .4, Very Good = .2, Excellent = 0	0.503
18. Hearing problems	Legally deaf = 1, Poor = .8, Fair = .6, Good = .4, Very Good = .2, Excellent = 0	0.441
**Medical symptoms during the past 2 years**		
19. Severe fatigue	Yes = 1, No = 0	0.285
20. Panting, cough or phlegm	Yes = 1, No = 0	0.236
21. Involuntary urine loss	Yes = 1, No = 0	0.107
22. Leg pain on walking	Yes = 1, No = 0	0.476
23. Stomach pain, indigestion or diarrhea	Yes = 1, No = 0	0.214
24. Bodily pain	Frequent and severe = 1, Frequent and moderate = 2/3, Frequent and Mild = 1/3, Not frequent = 0	0.298
25. Depressive symptoms	One ninth for each positive answer if the participant felt: depressed, unhappy, lonely, tired, sad, did not enjoy life, had no energy, restless sleep, or thought that everything did was an effort. 0 = none of the above present	0.427
**Activities of daily living**		
26. Difficulty with mobility	One fourth for positive answer if the participant had difficulty: picking up a 1-peso coin from the table, dressing including putting on shoes and socks, walking several blocks, walking across a room. 0 = none of the above	0.233
27. Difficulty with taking a shower	Yes = 1, No = 0	0.105
28. Difficulty with eating	Yes = 1, No = 0	0.119
29. Difficulty with getting in and out of bed	Yes = 1, No = 0	0.074
30. Difficulty with going to the toilet	Yes = 1, No = 0	0.033
**Instrumental activities of daily living**		
31. Difficulty with meal preparation	Yes = 1, No = 0	0.083
32. Difficulty with shopping	Yes = 1, No = 0	0.065
33. Difficulty with taking medications	Yes = 1, No = 0	0.051
34. Difficulty with handling own finances	Yes = 1, No = 0	0.054

† Point estimate for the older Mexican adults aged 65 and plus

**Figure 2:**
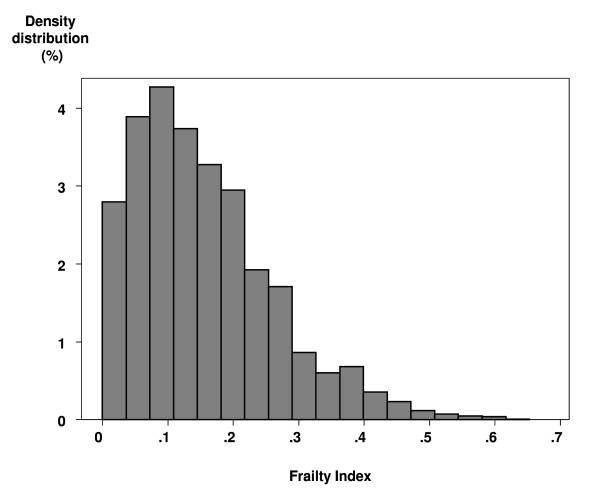
**Distribution of the fraility index in the Mexican elderly population**. Data from the Mexican Health and Ageing Study, 2001. Sample weighted estimates.

The constructed frailty index had an average value of .16 in the target population (standard deviation = .11), ranging in the sample from 0 to .65 and showing a right-skewed distribution (Figure 2).

**Figure 3:**
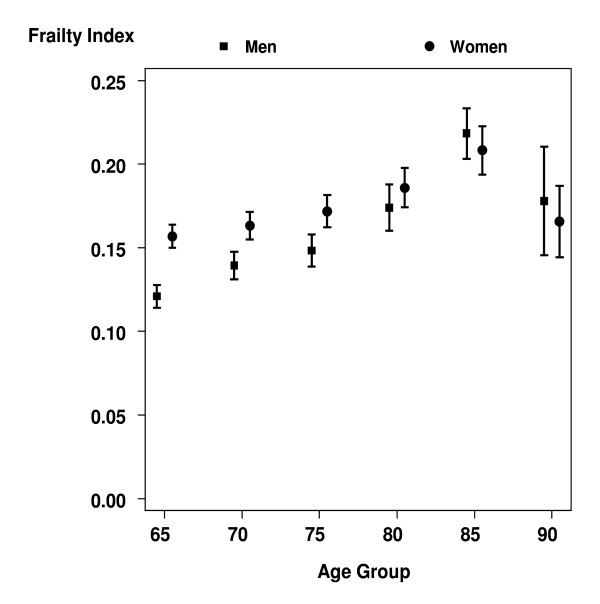
**Mean fraility index (and 95% CIs) by age and gender**. Sample weighted estimates.

In addition, the mean frailty index increased with age between the ages of 65 to 89 and then decreased in those aged 90 and older (Figure 3). This age effect was more clearly seen in men. In fact, women showed significantly higher mean frailty index values than men in the age groups younger than 80 years.

**Figure 4:**
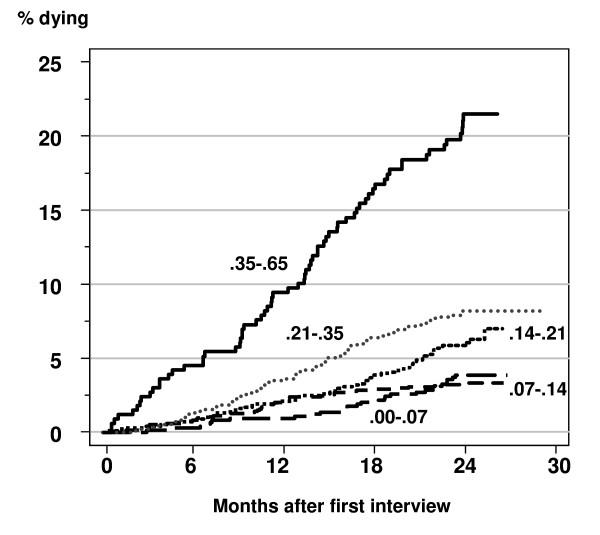
**Cumulative mortality by level of the fraility index for an average analyzed participant with respect to age (73 years) and gender (52.5% females)**. Estimates adjusted for age and gender.

The following five frailty index levels were obtained by the minimum variance method (with percentages of the Mexican elderly population shown in parenthesis): .00-.07 (17.4%), .07-.14 (30.8%), .14-.21 (24%), .21-.35 (21.4%), .35-.65 (6.5%). Figure 4 shows the cumulative percentage of persons dying during the follow-up by frailty level for an average Mexican with respect to age (73 years) and gender (52.5% females). Over the follow-up period, mortality in older Mexican adults increased with the frailty index level, especially in those with frailty index levels between .21 to less than.35 and those with frailty index levels between .35 and .65, reaching at 2 years of follow-up approximately 17% and 21%, respectively.

These findings were confirmed in the Cox proportional hazards models (Table 2). In all participants those in the two highest frailty levels have age and gender adjusted death hazards 2.2 and 6.45 times higher than the observed in the lowest frailty level. Women experience a 33% lower instantaneous risk of death than that observed in men and each year of increase in age is associated with a 5% increase in the risk of death. Cox models fitted separately in each gender show that in men there is a statistically significant increase in the age adjusted death hazard in both those with frailty index levels between .21 to less than.35 and those with frailty index levels between .35 and .65. In contrast, women show only a statistically significant increase in the age adjusted death hazard in the highest frailty index level (.35-.65).

**Table 2:** Mortality hazard ratios (and 95% CIs) for different levels of the frailty index, adjusted for covariates and stratified by gender.

	**All** **(n = 4082)**	**Men** **(n = 1932)**	**Women** **(n = 2150)**
**Frailty index**			
.00-.07	1	1	1
.07-.14	**0.93** (0.58-1.50)	**0.99** (0.56-1.76)	**0.83** (0.35-1.95)
.14-.21	**1.56** (1.00-2.44)	**1.30** (0.73-2.32)	**1.84** (0.85-3.96)
.21-.35	**2.20** (1.42-3.41)	**2.69** (1.59-4.57)	**1.73** (0.80-3.77)
.35-.65	**6.45** (4.10-10.14)	**5.96** (3.32-10.72)	**6.63** (3.07-14.35)
**Age (years)**	**1.05** (1.04-1.07)	**1.05** (1.02-1.07)	**1.06** (1.04-1.09)
			
**Gender**			
Men	1		
Women	**0.66** (0.52-0.84)		

Estimates Obtained through Cox regression.

**Figure 5:**
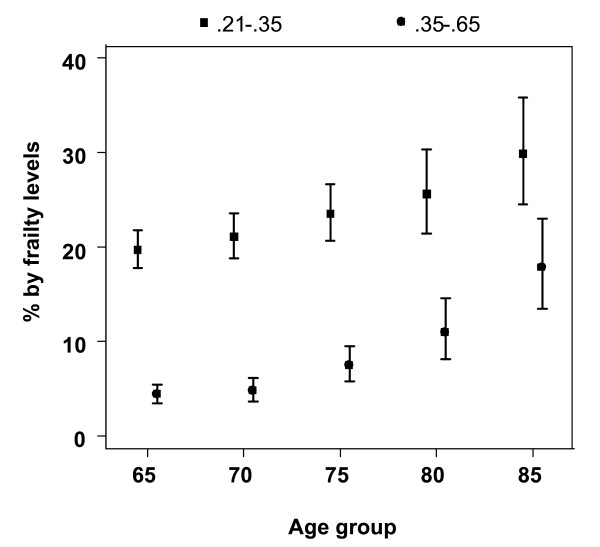
**Estimated percentage of Mexicans, aged 65 and older, with frailty index levels associated to excess mortality, by age group**.

Finally, figure 5 shows the prevalence of frailty index levels associated to increased mortality hazards in the Cox models by age group in the non-institutionalized Mexican elderly population. Both frailty levels show an increasing prevalence with age, especially after age 70. Elderly Mexicans with frailty index between .21 and less than .35 represent 19.7% of those aged 65-69 years and 29.8% of those aged 85 and older. More marked increases in the prevalence of persons with frailty index values .35 or higher are seen. These frailty index values are seen in 4.3% of those aged 65-69 years and 17.7% of those aged 85 and older. Thus, the proportion of Mexican older adults with frailty index levels associated to increased mortality (.21 and higher) represent 24.0% of those aged 65-69 years and 47.6% of those aged 85 and older.

## Discussion

This paper presents the results of an analysis of a frailty index, based on self report accounts of deficit accumulation and developed by Mitnitski and Rockwood [9], as an independent predictor of mortality in a probabilistic sample of non-institutionalized Mexican older adults, aged 65 and older. 92% of analyzed participants were followed up, for an average time of almost 2 years. This implies that we evaluated mortality over a relatively short period after the initial interview.

Interviews were conducted face to face by trained personnel and only those directly administered were analyzed here. We did not analyze data from 449 of the 4872 initially interviewed participants (9.2%) because a proxy had answered a questionnaire with insufficient data to construct the frailty index. These participants had a higher mortality than those directly interviewed and were likely to be very ill. Therefore, excluding them from our analysis could have weakened the association we found between the frailty index and mortality. In addition, the prevalence estimates of high frailty levels we report may be an underestimate of the values we could have found had we not excluded those with proxy questionnaires.

In spite of the above caveats, our study replicates previous findings from secondary analysis of similar datasets from North American and European developed countries [17], as well as from China [21,22], in institutional and community elderly using roughly equivalent versions of the deficit accumulation frailty index. In our analysis the frailty index showed a skewed distribution which, as previously found by Rockwood et al [23], could be fitted by a gamma distribution and a maximum value of .65 was observed, as others have also found [24,25].

As has been previously reported in other settings[9,15,17,26], both the mean frailty index and the prevalence of high frailty levels were found to increase with age in our sample of Mexican elderly, with some leveling off at very advanced ages possibly explained by selection of robust individuals at those ages [27,28]. In addition, we found that mean frailty levels were higher in women than in men before age 85, as other studies have also found [29]. The fact that women seem to accumulate more deficits, as measured by the frailty index, at a faster speed in late life may be explained by their higher rate of objective disability [1,30], possibly caused by genetic [27] and social factors [31], but also by better reporting of medically diagnosed conditions in women due to their greater access to health care [32].

In our analysis, the categorized frailty index strongly predicted 2-year death, independently of chronological age, in those with a frailty index with a value of at least .21, particularly in men, a finding consistent with previous studies [9,15,21,22,33,34].

This concept appears applicable to populations with diverse ethnicity and life styles. However, it would be important to study populations from developing countries with socioeconomic deprivation in order to validate the construct.

Regardless of how frailty is conceptualized and measured, vulnerability towards death has been explained by two biological mechanisms: allostatic load and sarcopenia. Seeman [35] defines allostatic load as the cumulative physiologic toll exacted on the body over time by efforts to adapt to life experiences. As an individual age he or she experiences complex losses in resting dynamics and maladaptive responses to perturbations [36]. This process starts likely early in life and this is the reason why we included serious health problems experienced before age 10 in our frailty index. Participants who reported a serious event during childhood can be defined as survivors. Because of little is known about the effect of early life events on aging course, these findings will mark further research.

Sarcopenia refers to the substitution of muscle by fat tissue, accelerated by physical inactivity, chronic diseases and disability, among other factors and has been found related to several adverse health outcomes [37]. In fact, studies using Fried's frailty definition have found that frail older adults have lower muscular density and muscle mass and higher fat mass than non frail elderly [38]. Beyond to this two biological explanations, it is possible to say that a combination of several factors such a lifestyles, social networks, economic status, emotional life are frequently behind of an specific accumulation of deficits [39]

Women showed a weaker association between the frailty index and the risk of dying over the next two years, as has also been found elsewhere [15,22,27]. Whether this is explained by a true biological difference [40] or, as stated above, by differential measurement error of a frailty index based on self report is a matter that needs to be further addressed.

To our knowledge this is the first study providing data on the magnitude of frailty in a Latin-American country and of its impact on mortality. We found that one in four Mexicans aged 65 to 69 years and almost one in two Mexicans aged 85 and older had frailty levels that increased their risk of dying over the next two years. This has obvious health care implications in the light of studies showing that frailty is a dynamic process, characterized by frequent transitions to states of lower frailty and therefore suggesting opportunities not only for preventing but for alleviating frailty [27,41].

## Conclusion

A frailty index based on a simple count of self reported deficits offering an adequate prediction of mortality, both in clinical and epidemiological settings, is a useful tool to deal with frailty and its consequences, especially in developing countries.

## Competing interests

The authors declare that they have no competing interests.

## Authors' contributions

JJGG design the study, prepared the database and contribute to the analysis. CGP collaborated with the design, the analysis and prepare the draft. FFM contribute to the conception of the study and carried out the analysis, interpreted the data and review the manuscript. LMGG was involved in the design of the study, the interpretation of the data and the revision of the manuscript. All gave final approval of the submitted version.

## Pre-publication history

The pre-publication history for this paper can be accessed here:


